# Investigation of the impact of machine operating parameters on beam delivery time and its correlation with treatment plan characteristics for synchrotron-based proton pencil beam spot scanning system

**DOI:** 10.3389/fonc.2022.1036139

**Published:** 2022-11-10

**Authors:** Xiaoying Liang, Chris Beltran, Chunbo Liu, Jiajian Shen, Martin Bues, Keith M. Furutani

**Affiliations:** ^1^ Department of Radiation Oncology, Mayo Clinic Florida, Jacksonville, FL, United States; ^2^ Department of Radiation Oncology, The First Affiliated Hospital of Zhengzhou University, Zhengzhou, China; ^3^ Department of Radiation Oncology, Mayo Clinic Arizona, Phoenix, AZ, United States

**Keywords:** beam delivery time, proton pencil beam scanning, synchrotron, machine operation parameters, treatment plan characteristics.

## Abstract

**Purpose:**

To investigate the beam delivery time (BDT) reduction due to the improvement of machine parameters for Hitachi synchrotron-based proton PBS system.

**Methods:**

BDTs for representative treatment plans were calculated to quantitatively estimate the BDT improvement from our 2015 system at Mayo Clinic in Arizona to our system to be implemented in 2025 at Mayo Clinic in Florida, and to a hypothetical future system. To specifically assess how each incremental improvement in the operating parameters reduced the total BDT, for each plan, we simulated the BDT 10,368 times with various settings of the nine different operating parameters. The effect of each operating parameter on BDT reduction and its correlation with treatment plan characteristics were analyzed. The optimal number of multiple energy extraction (MEE) layers per spill for different systems was also investigated.

**Results:**

The median (range) decrease in BDT was 60% (56%-70%) from the 2015 to the 2025 system. The following incremental improvement in parameters of the 2015 system for the 2025 system played an important role in this decreased BDT: beam intensity (8 to 20 MU/s), recapture efficiency (50% to 80%), number of MEE layers per spill (4 to 8), scanning magnet preparation and verification time (1.9 to 0.95 msec), and MEE layer switch time (200 to 100 msec). Reducing the total spill change time and scanning magnet preparation and verification time from those of the 2025 system further reduced BDT in the hypothetical future system. 8 MEE layers per spill is optimal for a system with 50% recapture efficiency; 16 MEE layers per spill is optimal for a system with 80% recapture efficiency; and more than 16 MEE layers per spill is beneficial only for a system close to 100% recapture efficiency.

**Conclusions:**

We systematically studied the effect of each machine operating parameter on the reduction in total BDT and its correlation with treatment plan characteristics. Our findings will aid new and existing synchrotron-based proton beam therapy centers to make balanced decisions on BDT benefits vs. costs when considering machine upgrade or new system selection.

## 1 Introduction

Decreasing proton beam delivery time (BDT) is desirable because it reduces intrafractional patient movement (1–3). Decreasing BDT also increases clinical throughput, which is especially valuable for proton beam therapy. Although proton beam therapy has high therapeutic efficacy for certain types of tumors (4–8), only a small percentage of patients who receive radiotherapy are treated with proton beam therapy because of a limited number of proton beam therapy facilities (9, 10). Furthermore, a recent study (11) reported that treatment time affects the proton dose to the circulating blood and suggested that shorter BDTs can reduce the dose to the circulating blood. Lymphocytes are sensitive to radiation, and previous studies (12, 13) have shown that radiation-induced lymphopenia negatively affects clinical outcomes in patients with various cancer types.

Several studies have investigated means to reduce BDT *via* treatment planning optimization (11–16) or *via* advancing treatment delivery techniques (17, 18). A study by Younkin et al. (18) reported that BDT was decreased with the advanced beam extraction technique of multiple energy extraction (MEE) (19, 20) and that BDT can be further reduced by improving synchrotron operating parameters, such as the number of monitor units (MUs) per spill and recapture efficiency. During the past decade, efforts have been made to improve the hardware and control software in proton accelerators and beam extraction and delivery systems. Our group recently published a study on BDT of the novel continuous scanning mode (dose-driven continuous scanning [DDCS]) and found that DDCS was able to reduce BDT compared to conventional discrete spot scanning (DSS) (21). In the current study, we focus on investigating the BDT of DSS for systems with different operating parameters. As for today, clinically all the Hitachi proton PBS is delivered by DSS. Here, we systematically studied the effect of machine operating parameters on DSS BDT for the Hitachi (Hitachi, Ltd) synchrotron-based pencil beam spot scanning with MEE extractions. We aimed to identify the key operating parameter improvement that led to a reduction in BDT from our proton beam system established in 2015 to our system that will be clinically operational in 2025, as well as the potential key parameters for further reductions in BDT in a hypothetical future proton beam delivery system. We also assessed the correlation between the BDT reduction resulting from each improved operating parameter and treatment plan characteristics.

## 2 Method and materials

This study was approved by our institution’s institutional review board (IRB#21-012011).

### 2.1 Beam delivery time calculation

Software developed in-house was used for the BDT calculation. The BDT calculation algorithm and validation of the software are previously described (21). Briefly, the total BDT for discrete spot scanning can be calculated as:


(1) $$BDT=T_{stop}+T_{SSw}+T_{SCh}+T_{LSw}$$

*T*_*stop*_,*T*_*SSw*_,*T*_*SCh*_, and *T*_*LSw*_ represent the total stop time, total spot switch time, total spill change time, and total MEE layer switch time, respectively. In simplified terms, these variables can be expressed as:


(2) $$T_{stop}=\sum_{i=1}^{n}spot\;MU*beam\;intensity\;$$

(3) $$T_{SSw}=\;\sum_{i=1}^{n}\left(\frac{spot^{\prime }s\;distance}{scanning\;speed}+scanning\;magnet\;preparation\;and\;verification\;time\right)$$

(4) $$T_{Sch}=\;number\;of\;spill\;change*spill\;change\;time$$

(5) $$T_{LSw}=number\;of\;MEE\;extraction\;*layer\;switch\;time\;within\;MEE\;extraction$$

n is the number of spots in the plan.

### 2.2 Treatment plan selection

A total of 36 representative treatment plans, with 6 treatment sites (breast, central nervous system [CNS], head and neck, liver, lung, and prostate) and 6 patients per treatment site, were selected (**Table 1**). This set of representative clinical PBS plans were also used in our previous study (21). Using this same set, the current study and the previous published study (21) separately investigated the two distinct scanning modes in the treatment delivery. In our system, 1 MU is corresponding to about 0.7 billion of protons for intermediate proton energies (22).

**Table 1:** Treatment plan characteristics.

Treatment site/patient no.	Dose/fraction (GyRBE)	Plan MU	No. of layers	No. of spots^a^	Mean MUs per layer
Breast					
1	2.00	1,082.5	183	59,442	5.9
2	2.00	844.4	180	42,020	4.7
3	2.00	455.8	150	29,455	3.0
4	2.67	785.8	85	52,129	9.2
5	2.67	1,613.8	180	56,127	9.0
6	7.30	395.9	64	16,576	6.2
CNS					
1	1.20	14.2	136	1,437	0.1
2	1.80	42.3	149	4,162	0.3
3	1.80	86.3	131	10,575	0.7
4	1.80	813.3	286	46,288	2.8
5	6.00	161.6	111	11,140	1.5
6	7.00	169.1	126	8,447	1.3
Head and neck					
1	1.20	40.5	94	3,957	0.4
2	2.00	299.2	180	12,865	1.7
3	2.00	278.7	274	20,039	1.0
4	2.00	323.2	211	21,618	1.5
5	2.00	464.7	426	41,659	1.1
6	7.50	147.7	51	5,148	2.9
Liver					
1	2.00	158.2	118	17,032	1.3
2	2.50	118.7	124	30,149	1.0
3	3.67	134.0	125	20,564	1.1
4	3.87	286.7	99	23,107	2.9
5	8.00	255.0	89	11,583	2.9
6	10.00	438.9	110	46,787	4.0
Lung					
1	2.00	106.6	119	13,466	0.9
2	2.00	166.5	106	25,453	1.6
3	2.00	106.7	147	16,631	0.7
4	3.00	320.2	173	21,234	1.9
5	5.00	181.0	73	23,062	2.5
6	12.00	177.8	111	15,948	1.6
Prostate					
1	1.80	45.4	56	3,796	0.8
2	2.00	49.9	68	4,043	0.7
3	2.50	97.5	46	5,794	2.1
4	2.70	357.3	126	29,922	2.8
5	3.00	85.1	40	4,083	2.1
6	7.00	234.2	67	10,294	3.5

CNS, central nervous system; MU, monitor unit.

a For layer repainting cases, the number of spots was calculated as the summation of each spot multiplied by the number of repainting.

### 2.3 BDT simulations and analysis

The operating parameters for the proton beam system at Mayo Clinic in Arizona installed in 2015 (2015 system), the system to be installed at Mayo Clinic in Florida for clinical use in 2025 (2025 system), and a hypothetical future system with parameters improved from the 2025 system (future system) are listed in **Table 2**. These operating parameters include the number of MEE layers per spill, recapture efficiency, maximum number of MUs per spill, maximum extraction time per cycle, scanning magnet scan speed, beam intensity, spill change time, MEE layer switch time, and scanning magnet preparation and verification time. To quantitatively estimate the BDT improvement from the 2015 system to the 2025 system and the potential improvement in the future system, we calculated the BDTs for the 36 representative treatment plans with the three systems. The improvement in the total BDT and each of its components (total stop time, total spot switch time, total spill change time, and total MEE layer switch time) was calculated.

**Table 2:** Operating parameters of 2015, 2025, and hypothetical future synchrotron-based proton pencil beam spot scanning systems.

Parameter	2015	2025	Future	Various settings for 10,368 BDT simulations
No. of MEE layers per spill	4	16	Unlimited	4, 8, 16, Unlimited
Recapture efficiency, %	50	80	100	50, 80, 100
Maximum no. MUs per spill	20	30	60	20, 30, 60
Maximum extraction time per cycle, s	8	500	500	8, 500
Scanning magnet scan speed in [x,y] direction, m/s	[6,10]	[8,20]	[40,120]	[6,10], [8,20], [16,40], [40,120]
Beam intensity, MU/s	8	40	40	8, 20, 40
Spill change time, s	2	2	1	2, 1
MEE layer switch time, msec	200	100	100	200, 100
Scanning magnet preparation and verification time, msec	1.9	0.95	0	1.9, 0.95, 0

The various settings used for 10,368 BDTs simulations were also listed. MEE, multiple energy extraction; MU, monitor unit.

To specifically assess how each step of improvement in the operating parameters reduced the total BDT, we simulated the BDTs with different combinations of settings of the nine different operating parameters (the last column of **Table 2**), with each setting representing an incremental improvement from that of the 2015 system. For each plan, the number of simulations was the product of the number of chosen steps for each operating parameter, which resulted in 10,368 simulations. The set of simulated BDTs were separated into subsets by each dependent parameter to enable univariable analyses. For example, to study the effect of improving the MEE layer switch time for each plan, the 10,368 BDTs were grouped into one subset of 5,184 BDTs with an MEE layer switch time of 200 msec and one subset of 5,184 BDTs with an MEE layer switch time of 100 msec. This grouping method was then repeated for each of the nine operating parameters. Correlations between the BDT reduction resulting from each improved operating parameter and treatment plan characteristics were determined with R^2^ (also referred to as the coefficient of determination).

## 3 Results

### 3.1 BDT reduction from the 2015 system to the 2025 system, and possible further reduction in a hypothetical future system

**Figure 1:**
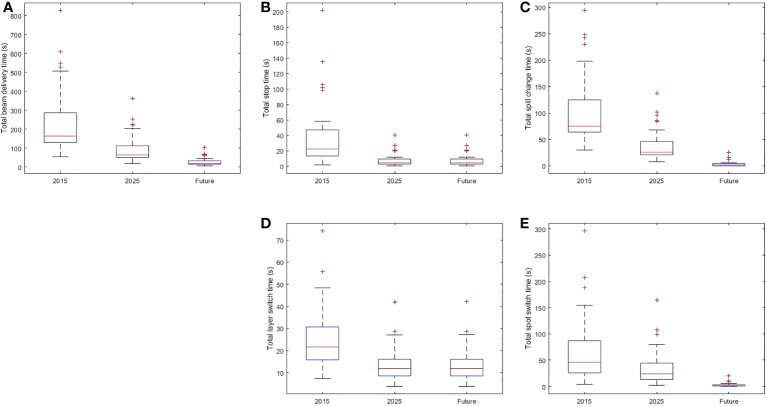
Box Plot of Beam Delivery Time and Time of Each BDT Component in the 2015, 2025, and Future Systems. **(A)** The distribution of total beam delivery time. **(B)** The distribution of the total stop time. **(C)** The distribution of the total spill change time. **(D)** The distribution of the total layer switch time. **(E)** The distribution of the spot switch time. Within each box, horizontal central line represents the median value and box represents the 25^th^ to 75^th^ percentile.

**Supplementary Table 1** list the BDTs for the 36 representative treatment plans using operating parameters of the 2015, 2025, and future systems. Box plots of the total BDTs and the time of its each component (total stop time, total spill change time, total layer switch time, and total spot switch time) of the 2015, 2025, and future system is shown in **Figure 1**. The median (range) decrease in total BDT from the 2015 to 2025 system was 60% (56%-70%). The total stop time was reduced by 80% for all treatment plans because the beam intensity improved from 8 to 40 MU/s. The median (range) reduction in total spill change time was 65% (53%-81%) because of the reduced number of spill changes resulting from a combination of an increased number of MEE layers per spill from 4 to 16, recapture efficiency from 50% to 80%, number of MUs per spill from 20 to 30, and maximum extraction time per spill from 8 seconds to 500 seconds. The median (range) reduction in total MEE layer switch time was 48% (35%-50%) because of the decreased time for each MEE layer switch (from 0.2 second to 0.1 second). The median (range) reduction in total spot switch time was 49% (45%-50%) because of the increased scanning magnet scan speed and decreased scanning magnet preparation and verification time. In the future system, the median (range) decrease in the total BDT from the 2025 system was 70% (50%-78%). The median (range) reduction in total spill change time was 93% (82%-100%) because of the decreased each spill change time and increased number of MUs per spill, number of MEE layers per spill, and recapture efficiency. The median (range) reduction in the total spot switch time was 94% (88%-98%) because of the decreased scanning magnet preparation and verification time and increased scanning speed.

**Figure 2:**
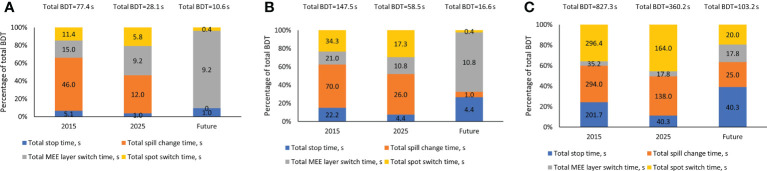
Contribution of Each Beam Delivery Time (BDT) Component for 3 Representative Treatment Plans. **(A)** Representative treatment plan for patient 1 in the head and neck cohort (1.2 GyRBE/fx; 3,957 spots; 0.4 mean MUs per layer; and total plan MU of 40.5). **(B)** Representative treatment plan for patient 6 in the lung cohort (12 GyRBE/fx; 15,948 spots; 1.6 mean MUs per layer; and total plan MU of 177.8). **(C)** Representative treatment plan for patient 5 in the breast cohort (2.67 GyRBE/fx; 56,127 spots; 9.0 mean MUs per layer; and total plan MU of 1,613.8). All treatment plan characteristics are described in **Table 1**. MEE indicates multiple energy extraction.

Each component of the total BDT of three representative treatment plans is displayed in **Figure 2**. The 3 representative treatment plans were: patient 1 in the head and neck cohort, patient 6 in the lung cohort, and patient 5 in the breast cohort (**Table 1**). These treatment plans were chosen because they represent low, medium, and high values for the total number of MUs, mean number of MUs per layer, and number of spots. These 3 plans also represent low, medium, and high prescription dose per fraction. As shown in **Figure 2**, every component of the total BDT was decreased from the 2015 system to the 2025 system. However, the total spill change time remained the largest component of the total BDT in both the 2015 and 2025 systems, except for extra-large targets such as bilateral breast plus regional lymph nodes case. Therefore, decreasing the total spill change time effectively further reduced the total BDT in the future system.

### 3.2 Effect of each operating parameter on BDT and correlation with treatment plan characteristics

The following improved operating parameters played an important role in the decrease in total BDT from the 2015 to the 2025 system (**Table 3**): increased beam intensity from 8 to 20 MU/s (mean reduction in BDT, 17%), increased recapture efficiency from 50% to 80% (mean reduction in BDT, 16%), increased number of MEE layers per spill from 4 to 8 (mean reduction in BDT, 13%), decreased scanning magnet preparation and verification time from 1.9 msec to 0.95 millisecond (mean reduction in BDT, 14%), and decreased MEE layer switch time from 200 to 100 msec (mean reduction in BDT, 12%). In the future system, decreasing the spill change time from 2 seconds to 1 second and decreasing the effective scanning magnet preparation and verification time to 0 resulted in a mean reduction in BDT of 23% and 17%, respectively.

**Table 3:** Mean beam delivery time (BDT) percentage reduction with improved operating parameters for 36 representative treatment plans.

Treatment site/Patient no.	Dose/fx,GyRBE	No. of MEE layers per spill			Recapture efficiency, %		Number of MUs per spill		Max extraction time, s	Beam intensity, MU/s		Scanning magnet scan speed in [x,y] direction, m/s			Spill change time, s	Layer switch time, s	Scanning magnet preparation and verification time, s	
		4 to 8	8 to 16	16 to unlimited	50 to 80	80 to 100	20 to 30	30 to 60	8 to 500	8 to 20	20 to 40	[6,10]to [8,20]	[8,20] to [16,40]	[16,40] to [40,120]	2 to 1	0.2 to 0.1	1.9E-03 to 9.5E-04	9.5E-04 to 0
**Breast**																		
1	2.00	3	1	0	12	7	8	10	5	25	11	13	8	6	19	6	16	19
2	2.00	4	1	0	12	8	8	8	4	23	10	16	11	8	19	7	14	16
3	2.00	8	2	1	15	9	7	7	4	21	9	10	7	5	21	9	16	19
4	2.67	1	0	0	10	6	9	10	5	26	11	11	7	5	18	4	20	25
5	2.67	2	0	0	8	5	8	11	5	26	12	16	13	10	18	4	12	13
6	7.30	3	0	0	14	9	12	10	7	29	13	4	3	2	22	7	15	18
**CNS**																		
1	1.20	32	19	9	15	4	1	1	3	2	1	1	1	1	30	24	3	3
2	1.80	28	14	8	17	6	1	3	3	5	2	3	2	1	29	21	6	7
3	1.80	21	9	4	17	8	3	4	3	10	3	6	4	2	25	16	13	15
4	1.80	9	2	0	15	9	8	7	4	21	9	10	6	4	22	10	14	17
5	6.00	17	6	2	18	9	4	6	4	16	7	4	3	2	24	14	13	16
6	7.00	17	6	3	20	10	5	6	4	16	7	4	3	2	26	15	10	11
**HN**																		
1	1.20	24	11	6	18	7	3	3	4	7	3	4	3	2	28	19	8	9
2	2.00	15	5	2	18	9	7	6	3	19	8	6	4	3	25	14	10	11
3	2.00	19	7	3	18	8	4	5	3	14	5	6	4	3	25	15	11	13
4	2.00	16	5	2	18	8	5	6	4	17	7	5	4	2	24	13	13	15
5	2.00	17	7	3	16	8	4	5	3	13	5	7	5	4	24	14	13	15
6	7.50	9	3	0	18	11	8	7	6	25	10	5	4	3	24	11	11	12
**Liver**																		
1	2.00	14	5	2	17	7	5	4	3	15	5	9	6	4	22	12	17	20
2	2.50	13	6	2	13	6	3	3	2	9	3	14	9	7	18	11	23	29
3	3.67	17	6	2	16	8	4	5	3	13	4	6	4	2	22	13	20	24
4	3.87	8	3	1	14	8	6	8	4	21	9	7	4	3	21	9	19	23
5	8.00	9	2	0	18	11	9	8	5	23	10	3	2	1	24	11	13	15
6	10.00	5	1	0	12	6	6	7	4	20	8	10	8	5	17	7	23	30
**Lung**																		
1	2.00	17	7	3	18	8	4	4	3	11	4	8	5	3	23	14	16	18
2	2.00	12	4	1	15	7	4	4	3	14	6	8	5	4	20	11	22	28
3	2.00	19	8	4	16	8	3	4	2	10	3	6	4	2	24	15	16	20
4	3.00	13	4	1	18	10	6	6	4	18	7	6	4	3	23	12	14	16
5	5.00	8	2	0	16	8	7	5	4	17	8	6	4	3	19	9	23	30
6	12.00	14	6	1	18	10	5	6	4	17	7	2	1	1	23	13	17	21
**Prostate**																		
1	1.80	22	11	4	18	7	5	5	5	11	4	4	3	2	25	17	12	13
2	2.00	24	9	5	18	7	4	4	5	11	4	4	3	2	26	17	11	12
3	2.50	14	4	1	15	10	8	5	6	20	8	7	4	3	23	12	14	16
4	2.70	8	2	1	15	9	6	7	4	20	8	9	5	4	20	9	19	23
5	3.00	12	4	1	20	8	9	8	7	22	8	6	4	3	23	12	12	14
6	7.00	8	4	1	13	10	10	9	5	25	11	6	3	2	23	9	13	16

Color scale depicts the extent of BDT reduction, in which green indicates the maximum reduction in BDT and red indicates the minimum reduction in BDT. Gray cells indicate the changes in the hypothetical future system. CNS, central nervous system; MEE, multiple energy extraction; MU, monitor unit.

#### 3.2.1 Number of MEE layers per spill

**Figure 3:**
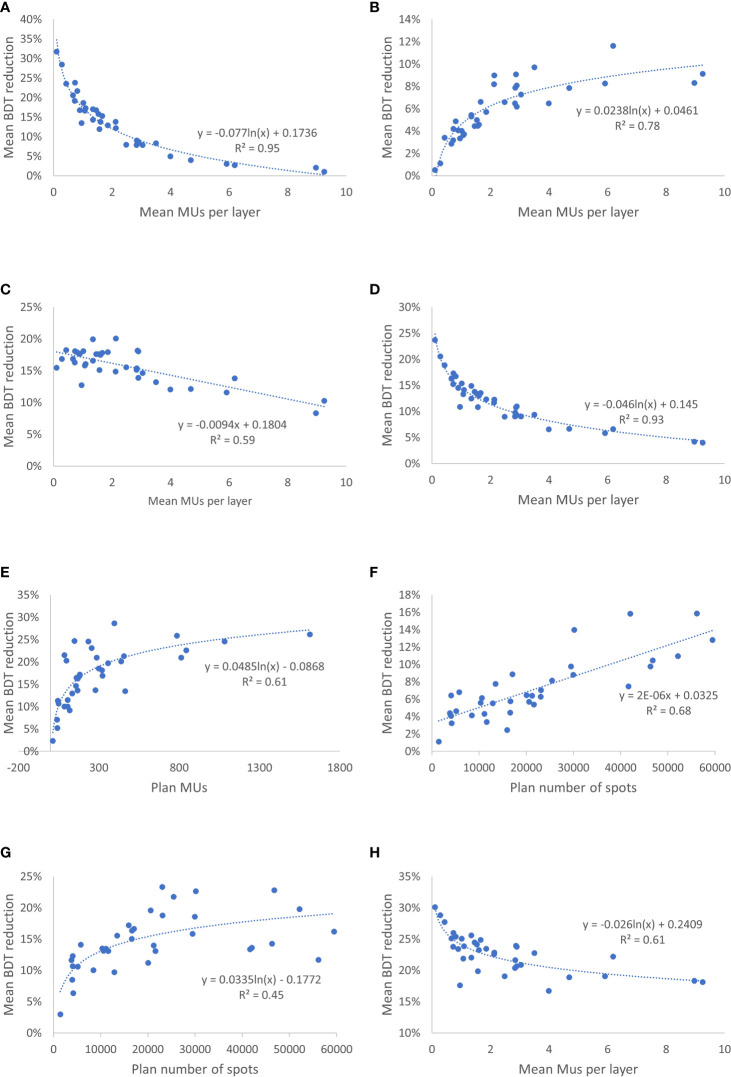
Correlation Between Mean Reduction in Beam Delivery Time (BDT) From Improved Operating Parameters and Treatment Plan Characteristics. **(A)** Mean reduction in BDT from increasing the number of multiple energy extraction (MEE) layers per spill from 4 to 8 as a function of the treatment plan mean number of monitor units (MUs) per layer. **(B)** Mean reduction in BDT by increasing the number of monitor unit (MU) per spill from 20 to 30 as a function of the treatment plan mean number of MUs per layer. **(C)** Mean reduction in BDT by increasing the recapture efficiency from 50% to 80% as a function of the treatment plan mean number of MUs per layer. **(D)** Mean reduction in BDT by reducing the layer switch time from 200 to 100 msec as a function of the treatment plan mean number of MUs per layer. **(E)** Mean reduction in BDT by increasing the beam intensity from 8 to 20 MU/s as a function of the treatment plan number of MUs. **(F)** Mean reduction in BDT by increasing the scanning magnet scan speed from [6,10] to [8,20] m/s in the [x,y] direction as a function of the treatment plan spot number. **(G)** Mean reduction in BDT by reducing the scanning magnet preparation and verification time from 1.9 msec to 0.95 msec as a function of the treatment plan spot number. **(H)** Mean reduction in BDT by reducing the spill change time from 2 seconds to 1 second as a function of the treatment plan mean number of MUs per layer.

Changing the number of MEE layers per spill from 4 to 8 had a greater effect on the total BDT reduction than did changing it from 8 to 16 or from 16 to unlimited (**Table 3**). The mean reduction in BDT from increasing the number of MEE layers per spill from 4 to 8 was strongly correlated with the treatment plan mean number of MUs per layer (R^2^ = 0.95), which resulted in an exponentially increased reduction in BDT with a decreasing mean number of MUs per layer (**Figure 3A**). For the treatment plan with the smallest mean number of MUs per layer (mean MUs per layer, 0.1; patient 1 in the CNS cohort), the mean reduction in BDT was 32% when the number of MEE layers per spill increased from 4 to 8 (**Table 3**). Increasing the number of MEE layers per spill from 8 to 16 had a small effect on the mean reduction in BDT for most treatment plans. Only treatment plans with a small mean number of MUs per layer had a mean reduction in BDT greater than 10%. For example, the treatment plans for patients 1 and 2 in the CNS cohort and patient 1 in the head and neck cohort (mean MUs per layer of 0.1, 0.3, and 0.4, respectively, **Table 1**), had a mean reduction in BDT greater than 10% (**Table 3**). Further increasing the number of MEE layers per spill above 16 had a minimal effect on the BDT for all the treatment plans. In addition, increasing the number of MEE layers per spill had a much lower effect on the reduction in BDT for the breast treatment plans than it did for the other treatment sites because of their larger mean number of MUs per layer.

#### 3.2.2 Number of MUs per spill

Increasing the MU per spill from 20 to 30 or doubling it from 30 to 60 resulted in a small mean reduction in BDT that ranged from 1% to 12% and 1% to 11%, respectively (**Table 3**). The mean reduction in BDT from increasing the number of MUs per spill had a strong logarithmic correlation with the treatment plan mean number of MUs per layer (R^2^ = 0.78) (**Figure 3B**).

#### 3.2.3 Recapture efficiency

Improving the recapture efficiency from 50% to 80% resulted in a mean reduction in BDT that ranged from 8% to 20% for all treatment plans (**Table 3**). Further improving the recapture efficiency from 80% to 100% resulted in a further reduction in BDT, which ranged from 4% to 11% (**Table 3**). The mean reduction in BDT from increasing the recapture efficiency had a negative linear correlation with the treatment plan mean number of MUs per layer (R^2^ = 0.59) (**Figure 3C**).

#### 3.2.4 Maximum extraction time per spill

Increasing the maximum extraction time per spill from 8 seconds to 500 seconds had a minimal effect on the mean reduction in BDT that ranged from 2% to 7% (**Table 3**).

#### 3.2.5 MEE layer switch time

Improving the MEE layer switch time from 200 msec to 100 msec resulted in a small mean reduction in BDT for the breast treatment plans (4%-9%) and a moderate to large mean reduction in BDT for the CNS (10%-24%), head and neck (11%-19%), liver (7%-13%), lung (9%-15%), and prostate (9%-17%) treatment plans (**Table 3**). The effect of decreasing the MEE layer switch time from 200 msec to 100 msec on the mean reduction in BDT was strongly correlated with the treatment plan mean number of MUs per layer (R^2^ = 0.93) (**Figure 3D**).

#### 3.2.6 Beam intensity

Increasing the beam intensity from 8 MU/s to 20 MU/s resulted in a moderate to large mean reduction in BDT (up to 29%) for most of the plans (**Table 3**). Further increasing the beam intensity from 20 to 40 MU/s resulted in a further small mean reduction in BDT ranged from 1% to 13%. The mean reduction in BDT from increasing the beam intensity was correlated with the treatment plan number of MUs (R^2^ = 0.61) (**Figure 3E**).

#### 3.2.7 Scanning magnet scan speed

Increasing the scanning magnet scan speed had a small effect for most of the plans (1%-16%, 1%-13%, and 1%-10%, respectively, for the three incremental scanning speed increasing, **Table 3**). The mean reduction in BDT from increasing the scanning magnet scan speed had positive linear correlation with the treatment plan number of spots (R^2^ = 0.68) (**Figure 3F**).

#### 3.2.8 Scanning magnet preparation and verification time

Decreasing the scanning magnet preparation and verification time from 1.9 to 0.95 millisecond and from 0.95 msec to 0 resulted in a mean reduction in BDT that ranged from 3% to 23% and 3% to 30%, respectively (**Table 3**). As expected, the mean reduction in BDT from the decreased scanning magnet preparation and verification time was correlated with the treatment plan number of spots (R^2^ = 0.45) (**Figure 3G**).

#### 3.2.9 Spill change time

Decreasing the spill change time from 2 seconds to 1 second resulted in a large mean reduction in BDT for all treatment plans (17%-30%) (**Table 3**). This reduction in BDT had a logarithmic correlation with the treatment plan mean number of MUs per layer (R^2^ = 0.61) (**Figure 3H**).

### 3.3 Optimal settings for the number of MEE layers per spill

**Figure 4:**
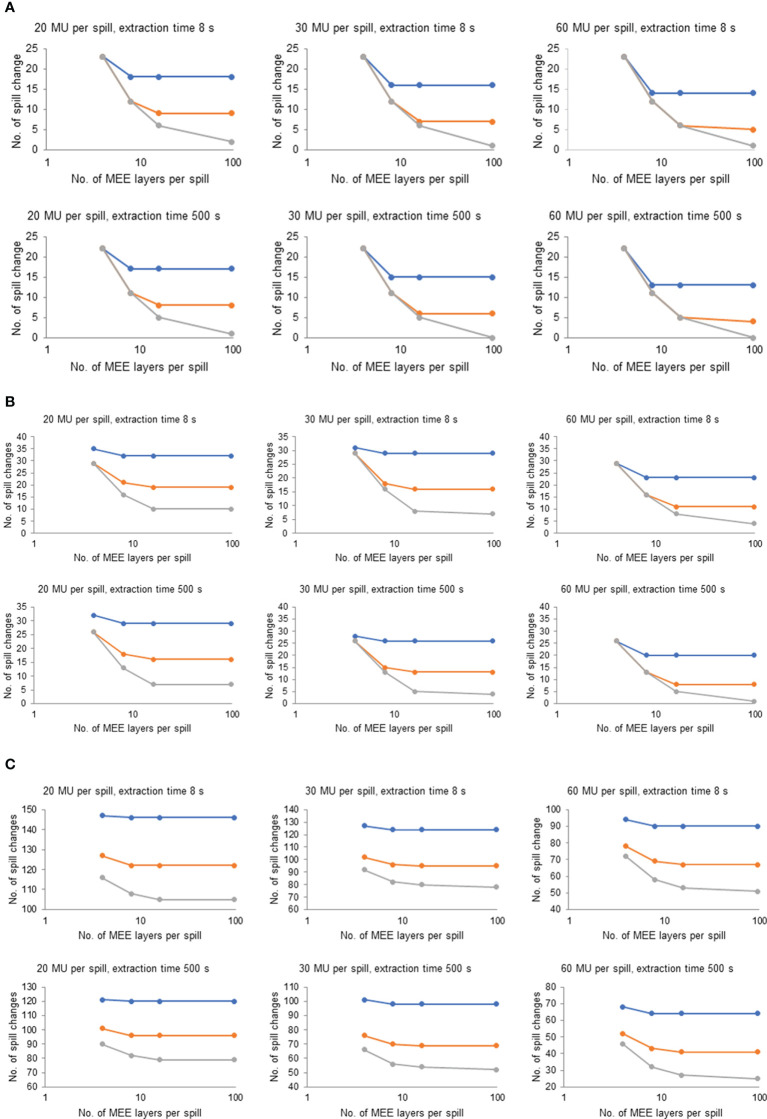
Effect of Number of Multiple Energy Extraction (MEE) Layers per Spill on the Number of Spill Changes With Various Recapture Efficiency, Monitor Unit (MU) per Spill, and Extraction Times for 3 Representative Treatment Plans. **(A)** Representative treatment plan for patient 1 in the head and neck cohort (1.2 GyRBE/fx; 3,957 spots; 0.4 mean MUs per layer; and total plan MU of 40.5). **(B)** Representative treatment plan for patient 6 in the lung cohort (12 GyRBE/fx; 15,948 spots; 1.6 mean MUs per layer; and total plan MU of 177.8). **(C)** Representative treatment plan for patient 5 in the breast cohort (2.67 GyRBE/fx; 56,127 spots; 9.0 mean MUs per layer; and total plan MU of 1,613.8). Blue line indicates 50% recapture efficiency; orange line, 80% recapture efficiency; and gray line, 100% recapture efficiency. All treatment plan characteristics are listed in **Table 1**.

Although there are large heterogeneities in plan characteristics among the 36 plans, the optimal number of MEE layers per spill was consistent for most of the plans and it appeared to depend only on recapture efficiency. **Figure 4** plots the number of spill changes as a function of the number of MEE layers per spill with different recapture efficiency, number of MUs per spill, and maximum extraction time values on the 3 representative treatment plans depicted in **Figure 2**. For a system with 50% recapture efficiency, 8 MEE layers per spill was optimal independent of the maximum extraction time and number of MUs per spill. For a system with 80% recapture efficiency, 16 MEE layers per spill was optimal regardless of the maximum extraction time and number of MUs per spill. Increasing the number of MEE layers per spill to more than 16 only worthwhile for a system with 100% recapture efficiency.

## 4 Discussion

The technologies used in proton therapy delivery systems are continuously advancing. We used a broad range of clinical proton beam plans in 6 different treatment sites with a range of prescription doses to quantitatively study the reduction in BDT resulting from improvements in the system operating parameters during the past 10 years and hypothetical improvements in future systems. The total BDT was substantially improved (60% decrease) from the 2015 system to the 2025 system, with each BDT component correspondingly decreased. For most treatment plans, the spill change time contribute most to the BDT, and the spot switch time contribute second to the BDT in the 2025 system. Therefore, effectively reduce the spill change time and the spot switch time will be able to further reduce the BDT in the future.

The overall sum total of BDTs for all the 36 plans was 139 minutes in the 2015 system, which reduced to 56 minutes in the 2025 system (**Supplementary Table 1**). Assuming an average of 30-minute schedule per patient, the saved 83 minutes on BDT can be used to treat three more patients and this translate to ~10% patient throughput increase. For a single accelerator multi-treatment room proton therapy center, the reduced BDT will shorten the waiting time when gantries are requesting beams back-to-back. This will lead to a further reduction of the patient on-table time, which improves patient comfortableness and further improves the patient throughput.

The BDT for a specific treatment plan depends on both the system operating parameters and plan characteristics. Detailed descriptions of the Hitachi proton beam systems and their specific operating parameters are previously reported (18, 23). The four BDT components (total stop time, total spot switch time, total MEE layer switch time, and total spill change time) were affected by different operating parameters. Therefore, the effect of each operating parameter on the reduction in total BDT depends not only on its effect on a specific BDT component but also on the contribution of its affected component to the total BDT. Consequently, most of the correlations between the effect of the operating parameters on BDT reduction and treatment plan characteristics were nonlinear.

The total stop time was specifically affected by beam intensity and the reduction in BDT from increased beam intensity was correlated with the treatment plan MUs. The operating parameters that affected the total spot switch time were the scanning magnet scan speed and scanning magnet preparation and verification time, but the latter had a greater effect on the BDT reduction. As expected, the effect of these operating parameters was correlated with the number of spots in the treatment plans. The total layer switch time was specifically affected by the MEE layer switch time. The effect of decreasing the MEE layer switch time on the reduction in BDT was correlated with the treatment plan mean number of MUs per layer. As observed in **Figure 2**, the total layer switch time occupied a bigger fraction of the total BDT for treatment plans with a smaller mean number of MUs per layer. Therefore, the effect of decreasing the MEE layer switch time on the total BDT was greater for plans with a smaller mean number of MUs per layer. The operating parameters that affect the number of spill changes are the number of MEE layers per spill, recapture efficiency, number of MUs per spill, and maximum extraction time per spill, and an interplay effect is present among these parameters. The maximum extraction time appeared to affect the number of spill changes the least, whereas the other three operating parameters were all correlated with the treatment plan mean number of MUs per layer. However, the effect of the number of MEE layers per spill and recapture efficiency on the mean reduction in BDT was negatively correlated with treatment plan mean number of MUs per layer, whereas the effect of the number of MUs per spill was positively correlated. The extractable charge per spill is more likely to be the limiting factor for a spill change in treatment plans with a large mean number of MUs per layer than for those with a small mean number of MUs per layer. Therefore, the effect of the number of MUs per spill is positive correlated with the treatment plan mean number of MUs per layer. Treatment plans with a small mean number of MUs per layer has a higher likelihood of extracting a greater number of layers per spill. Each layer extraction will cause a loss of a portion of extractable particles for a system with a recapture efficiency less than 100%. Therefore, the number of MEE layers per spill and the recapture efficiency are more important for treatment plans with a small mean number of MUs per layer than for those with a large mean number of MUs per layer. The spill change time remained as 2 seconds with no improvement from the 2015 system to the 2025 system, and the total spill change time contributed the most to the total BDT in both systems. Therefore, reducing the spill change time is an effective method to explore for further BDT reductions in future systems. Indeed, in the future hypothetical system, the spill change time reduced to 1 second, which resulted in a large reduction in BDT.

In the current study, we also investigated the optimal number of MEE layers per spill for the different systems. Among the 9 operating parameters, the number of MEE layers per spill is the only parameter that does not rely on the advancement of technology or a hardware upgrade because it is a user-defined parameter. Increasing the number of MEE layers per spill reduced the number of spill changes and thereby reduced BDT. However, when the number of MEE layers per spill was sufficiently large, other parameters (number of MUs per spill, or recapture efficiency, or maximum extraction time) became the limiting factor for the number of spill changes. Although having an unlimited number of MEE layers per spill do no harm on BDT, a higher number of MEE layers per spill will incur higher costs for a proton beam therapy center due to the vendor’s additional accelerator commissioning time. In addition, a higher number of MEE layers per spill requires additional beam time for physics calibration, validation, commissioning, and quality assurance. Therefore, determining the optimal number of MEE layers per spill for proton beam systems is valuable. Our study found that the optimal number of MEE layers per spill appeared to depend only on recapture efficiency. 8 MEE layers per spill is optimal for a system with 50% recapture efficiency; 16 MEE layers per spill is optimal for a system with 80% recapture efficiency; and more than 16 MEE layers per spill is beneficial only for a system approaching 100% recapture efficiency.

We would like to note that in this study, the MU was delivered by standard nonoptimized scanning path, namely it delivered in a line-by-line pattern. A recent study (24) on cyclotron-based FLASH proton therapy has shown that the spot delivery order optimization was able to reduce the BDT, and hence achieved significantly higher dose rate compared with the standard nonoptimized line-by-line pattern. The spot delivery order is also relevant to our beam delivery sequence as it affects the spot switch time. However, as shown in **Figure 2**, the total spot switch time is only a small component of the total BDT. Therefore, the reduction in BDT resulting from spot delivery order optimization will not be significant. Nevertheless, as we stated earlier, minimizing BDT is desired, hence all potential solutions should be explored.

## 5 Conclusion

We quantitatively investigated reductions in BDT because of improvements in the machine operating parameters during the past decade in our Hitachi synchrotron-based proton beam therapy systems. In addition, we systematically studied the effect of each machine operating parameter on the reduction in total BDT and its correlation with treatment plan characteristics. Furthermore, we identified the optimal number of MEE layers per spill for different systems. Our findings will aid new and existing proton beam therapy centers with Hitachi synchrotron-based pencil scanning systems to make balanced decisions on the benefits vs. costs for machine upgrade or new system selection.

## Data availability statement

The original contributions presented in the study are included in the article/**Supplementary Material**. Further inquiries can be directed to the corresponding author.

## Author contributions

XL, CB, and KF conceived the idea. CL, XL, CB, and KF developed the software program. XL conducted the study and wrote the manuscript. All authors contributed to the article and approved the submitted version.

## Funding

This research did not receive any specific grant from funding agencies in the public, commercial, or not-for-profit sectors.

## Acknowledgments

Nisha Badders, PhD, ELS, Mayo Clinic, provided editorial suggestions on an earlier draft of the manuscript.

## Conflict of interest

The authors declare that the research was conducted in the absence of any commercial or financial relationships that could be construed as a potential conflict of interest.

## Publisher’s note

All claims expressed in this article are solely those of the authors and do not necessarily represent those of their affiliated organizations, or those of the publisher, the editors and the reviewers. Any product that may be evaluated in this article, or claim that may be made by its manufacturer, is not guaranteed or endorsed by the publisher.
