# Supplementary material for: Investigation of the impact of machine operating parameters on beam delivery time and its correlation with treatment plan characteristics for synchrotron-based proton pencil beam spot scanning system
Source: Front Oncol. 2022 Nov 10;12:1036139. doi: 10.3389/fonc.2022.1036139 (PMC9691263; doi:10.3389/fonc.2022.1036139)
Supplement: Supplementary file 1 [file Table_1.docx]

**Supplementary Table 1.** Beam Delivery Time and Time of Each BDT Component in the 2015, 2025, and Future Synchrotron-Based Pencil Beam Scanning Proton Beam Systems

|  | **Total BDT, s** | | |  | **Total stop time, s** | | |  | **Total spill change time, s** | | |  | **Total layer switch time, s** | | |  | **Total spot switch time, s** | | |
| --- | --- | --- | --- | --- | --- | --- | --- | --- | --- | --- | --- | --- | --- | --- | --- | --- | --- | --- | --- |
| **Treatment site/patient no.** | **2015** | **2025** | **Future** |  | **2015** | **2025** | **Future** |  | **2015** | **2025** | **Future** |  | **2015** | **2025** | **Future** |  | **2015** | **2025** | **Future** |
| Breast | | | | | | | | | | | | | | | | | | | |
| 1 | 608.6 | 254.2 | 69.9 |  | 135.3 | 27.1 | 27.1 |  | 230.0 | 102.0 | 16.0 |  | 36.0 | 18.1 | 18.1 |  | 207.3 | 107.1 | 8.8 |
| 2 | 526.6 | 223.9 | 61.4 |  | 105.6 | 21.1 | 21.1 |  | 198.0 | 86.0 | 12.0 |  | 35.4 | 18.0 | 18.0 |  | 187.7 | 98.8 | 10.3 |
| 3 | 308.2 | 126.1 | 34.7 |  | 57.0 | 11.4 | 11.4 |  | 130.0 | 52.0 | 5.0 |  | 29.0 | 14.8 | 14.8 |  | 92.2 | 47.9 | 3.5 |
| 4 | 423.2 | 175.7 | 45.2 |  | 98.2 | 19.6 | 19.6 |  | 154.0 | 68.0 | 12.0 |  | 16.6 | 8.3 | 8.3 |  | 154.4 | 79.7 | 5.3 |
| 5 | 827.3 | 360.2 | 103.2 |  | 201.7 | 40.3 | 40.3 |  | 294.0 | 138.0 | 25.0 |  | 35.2 | 17.8 | 17.8 |  | 296.4 | 164.0 | 20.0 |
| 6 | 190.4 | 72.7 | 22.9 |  | 49.5 | 9.9 | 9.9 |  | 88.0 | 36.0 | 6.0 |  | 12.4 | 6.2 | 6.2 |  | 40.6 | 20.6 | 0.9 |
| CNS | | | | | | | | | | | | | | | | | | | |
| 1 | 89.9 | 27.2 | 13.7 |  | 1.8 | 0.4 | 0.4 |  | 64.0 | 12.0 | 0.0 |  | 20.2 | 12.8 | 13.2 |  | 4.0 | 2.1 | 0.1 |
| 2 | 113.6 | 39.7 | 16.1 |  | 5.3 | 1.1 | 1.1 |  | 74.0 | 18.0 | 0.0 |  | 22.2 | 14.4 | 14.6 |  | 12.1 | 6.2 | 0.4 |
| 3 | 128.9 | 51.8 | 15.8 |  | 10.8 | 2.2 | 2.2 |  | 66.0 | 22.0 | 0.0 |  | 22.8 | 12.7 | 12.8 |  | 29.3 | 14.9 | 0.8 |
| 4 | 549.3 | 221.8 | 66.6 |  | 101.7 | 20.3 | 20.3 |  | 242.0 | 96.0 | 12.0 |  | 55.8 | 28.6 | 28.6 |  | 149.9 | 76.9 | 5.7 |
| 5 | 138.4 | 53.0 | 16.5 |  | 20.2 | 4.0 | 4.0 |  | 70.0 | 24.0 | 1.0 |  | 20.4 | 10.8 | 10.8 |  | 27.8 | 14.2 | 0.6 |
| 6 | 144.1 | 54.8 | 17.4 |  | 21.1 | 4.2 | 4.2 |  | 76.0 | 26.0 | 0.0 |  | 23.6 | 12.4 | 12.4 |  | 23.4 | 12.2 | 0.8 |
| Head and neck | | | | | | | | | | | | | | | | | | | |
| 1 | 77.4 | 28.1 | 10.6 |  | 5.1 | 1.0 | 1.0 |  | 46.0 | 12.0 | 0.0 |  | 15.0 | 9.2 | 9.2 |  | 11.4 | 5.8 | 0.4 |
| 2 | 231.1 | 89.3 | 30.0 |  | 37.4 | 7.5 | 7.5 |  | 120.0 | 42.0 | 3.0 |  | 32.4 | 17.6 | 17.7 |  | 41.3 | 22.3 | 1.8 |
| 3 | 300.1 | 117.4 | 38.3 |  | 34.8 | 7.0 | 7.0 |  | 156.0 | 52.0 | 2.0 |  | 48.4 | 27.1 | 27.2 |  | 60.9 | 31.3 | 2.1 |
| 4 | 273.2 | 107.6 | 34.8 |  | 40.4 | 8.1 | 8.1 |  | 136.0 | 48.0 | 4.0 |  | 37.8 | 20.9 | 20.9 |  | 59.0 | 30.6 | 1.8 |
| 5 | 507.4 | 204.1 | 63.5 |  | 58.1 | 11.6 | 11.6 |  | 248.0 | 84.0 | 5.0 |  | 74.2 | 41.9 | 42.1 |  | 127.1 | 66.5 | 4.8 |
| 6 | 85.5 | 32.8 | 11.3 |  | 18.5 | 3.7 | 3.7 |  | 42.0 | 16.0 | 2.0 |  | 10.0 | 5.0 | 5.0 |  | 15.1 | 8.1 | 0.6 |
| Liver | | | | | | | | | | | | | | | | | | | |
| 1 | 164.6 | 64.8 | 19.1 |  | 19.8 | 4.0 | 4.0 |  | 74.0 | 24.0 | 2.0 |  | 21.4 | 11.6 | 11.6 |  | 49.4 | 25.2 | 1.6 |
| 2 | 196.7 | 83.7 | 18.1 |  | 14.8 | 3.0 | 3.0 |  | 68.0 | 22.0 | 0.0 |  | 22.4 | 12.1 | 12.1 |  | 91.4 | 46.6 | 3.1 |
| 3 | 162.8 | 64.9 | 16.6 |  | 16.7 | 3.3 | 3.3 |  | 74.0 | 24.0 | 0.0 |  | 22.4 | 12.3 | 12.3 |  | 49.7 | 25.3 | 1.0 |
| 4 | 193.5 | 78.8 | 22.2 |  | 35.8 | 7.2 | 7.2 |  | 80.0 | 32.0 | 4.0 |  | 18.6 | 9.7 | 9.7 |  | 59.1 | 29.9 | 1.4 |
| 5 | 153.1 | 61.3 | 19.7 |  | 31.9 | 6.4 | 6.4 |  | 76.0 | 32.0 | 4.0 |  | 17.4 | 8.7 | 8.7 |  | 27.8 | 14.3 | 0.6 |
| 6 | 312.0 | 131.5 | 31.8 |  | 54.9 | 11.0 | 11.0 |  | 106.0 | 42.0 | 6.0 |  | 20.8 | 10.6 | 10.6 |  | 130.4 | 68.0 | 4.2 |
| Lung | | | | | | | | | | | | | | | | | | | |
| 1 | 137.0 | 53.5 | 15.4 |  | 13.3 | 2.7 | 2.7 |  | 64.0 | 20.0 | 0.0 |  | 21.8 | 11.7 | 11.7 |  | 37.8 | 19.1 | 1.1 |
| 2 | 174.4 | 72.0 | 18.3 |  | 20.8 | 4.2 | 4.2 |  | 68.0 | 24.0 | 2.0 |  | 20.4 | 10.6 | 10.6 |  | 65.2 | 33.2 | 1.6 |
| 3 | 157.4 | 64.8 | 18.1 |  | 13.3 | 2.7 | 2.7 |  | 76.0 | 26.0 | 0.0 |  | 25.6 | 14.4 | 14.4 |  | 42.5 | 21.7 | 1.0 |
| 4 | 252.5 | 99.4 | 30.0 |  | 40.0 | 8.0 | 8.0 |  | 120.0 | 44.0 | 3.0 |  | 33.6 | 17.3 | 17.3 |  | 58.9 | 30.1 | 1.7 |
| 5 | 149.4 | 61.4 | 14.7 |  | 22.6 | 4.5 | 4.5 |  | 58.0 | 22.0 | 2.0 |  | 14.2 | 7.1 | 7.1 |  | 54.6 | 27.8 | 1.0 |
| 6 | 147.5 | 58.5 | 16.6 |  | 22.2 | 4.4 | 4.4 |  | 70.0 | 26.0 | 1.0 |  | 21.0 | 10.8 | 10.8 |  | 34.3 | 17.3 | 0.4 |
| Prostate | | | | | | | | | | | | | | | | | | | |
| 1 | 55.2 | 19.6 | 6.8 |  | 5.7 | 1.1 | 1.1 |  | 30.0 | 8.0 | 0.0 |  | 9.6 | 5.4 | 5.4 |  | 10.0 | 5.1 | 0.3 |
| 2 | 64.5 | 23.3 | 8.1 |  | 6.2 | 1.2 | 1.2 |  | 36.0 | 10.0 | 0.0 |  | 11.6 | 6.6 | 6.6 |  | 10.7 | 5.5 | 0.3 |
| 3 | 70.9 | 27.2 | 7.3 |  | 12.2 | 2.4 | 2.4 |  | 34.0 | 12.0 | 0.0 |  | 8.4 | 4.4 | 4.4 |  | 16.3 | 8.3 | 0.5 |
| 4 | 257.1 | 105.4 | 27.7 |  | 44.7 | 8.9 | 8.9 |  | 106.0 | 42.0 | 4.0 |  | 23.8 | 12.4 | 12.4 |  | 82.6 | 42.0 | 2.3 |
| 5 | 60.0 | 20.1 | 6.3 |  | 10.6 | 2.1 | 2.1 |  | 30.0 | 8.0 | 0.0 |  | 7.4 | 3.8 | 3.8 |  | 11.9 | 6.2 | 0.4 |
| 6 | 131.1 | 50.5 | 15.1 |  | 29.3 | 5.9 | 5.9 |  | 62.0 | 24.0 | 2.0 |  | 12.2 | 6.5 | 6.5 |  | 27.7 | 14.2 | 0.8 |

Abbreviations: BDT, beam delivery time; CNS, central nervous system.

**
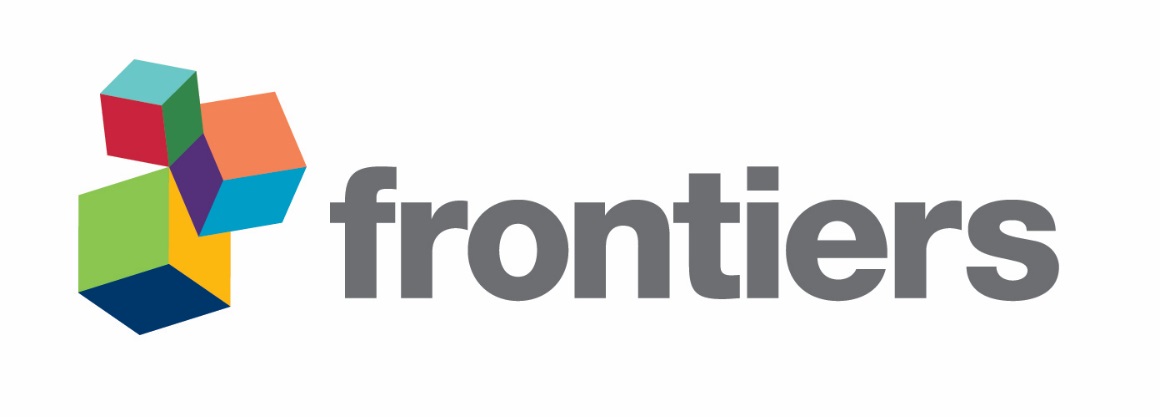
**
